# Treatment resistant psychosis in children and adolescents and clozapine: Nuances

**DOI:** 10.3389/fpsyt.2023.1014540

**Published:** 2023-02-24

**Authors:** Jigyansa Ipsita Pattnaik, Udit Kumar Panda, Suhas Chandran, Susanta Padhy, Jayaprakash Russell Ravan

**Affiliations:** ^1^Department of Psychiatry, Kalinga Institute of Medical Sciences, Bhubaneswar, Odisha, India; ^2^Child and Adolescent Psychiatry, St. Johns Medical College and Hospital, Bengaluru, India; ^3^Department of Psychiatry, All India Institute of Medical Sciences Bhubaneswar, Bhubaneswar, Odisha, India

**Keywords:** childhood psychosis, treatment resistance, childhood onset schizophrenia, early onset psychosis, early onset schizophrenia

## Abstract

With proliferation in research on high-risk psychosis spectrum diseases, it is crucial to distinguish a prodrome or psychosis-like episode in children and adolescents from true psychosis. The limited role of psychopharmacology in such circumstances is well-documented, underlining the difficulties in diagnosing treatment resistance. To add to the confusion is emerging data on the head-to-head comparison trials for treatment-resistant and treatment-refractory schizophrenia. Clozapine, the *gold-standard* drug for resistant schizophrenia and other psychotic psychopathology, lacks FDA or manufacturer guidelines for use in the pediatric population. Possibly due to developmental pharmacokinetic (PK) considerations, clozapine-related side effects are more commonly seen in children than adults. Despite evidence of an increased risk for seizures and hematological problems in children, clozapine is widely used off-label. Clozapine reduces the severity of resistant childhood schizophrenia, aggression, suicidality, and severe non-psychotic illness. There is inconsistent prescribing, administration, and monitoring of clozapine, and limited database evidence-backed guidelines. Despite the overwhelming efficacy, problems remain regarding unambiguous indications of use and risk-benefits assessments. This article reviews the nuances in the diagnosis of treatment resistance psychosis in childhood and adolescents and its management, in particular highlighting the evidence base for clozapine in this population group.

## 1. Introduction

The phrase “childhood-onset psychosis (COP)” was initially subsumed under the description of pervasive developmental disorder (PDD). In the DSM-V or ICD-11, the symptomatology and stated definitions/criteria of psychotic illnesses remain the same for children, adolescents, and adults (1, 2). Imaginative play and fantasy are frequent in young children and early teens. Children's imaginations and fantasy lives, combined with language and cognitive development stages, influence diagnostic accuracy, particularly when distinguishing between Childhood-Onset Schizophrenia (COS), Mood Disorders such as Bipolar Affective disorder (BPAD) or Major Depressive disorder (MDD), obsessive-compulsive disorder (OCD), and Attention-Deficit/Hyperactivity Disorders (ADHD) (1). The term “Psychosis Not Otherwise Specified (PNOS)” has been used to fill diagnostic gaps in instances with an ambiguous presentation. Neurocognitive issues are common in the absence of pathognomonic symptoms. Though there are no definite biochemical or neuroimaging indicators, psychosis may be an indication of widespread brain dysfunction. Pharmacotherapy for children and adolescents is primarily based on evidence from adult psychosis research. There has been little investigation in the domain of psychosocial and psychotherapy interventions for childhood psychosis as well (3).

As a result, it is important to first comprehend the idea of Childhood psychosis, followed by concerns about management. Clozapine's role in adults with treatment-resistant psychosis is well-established; however, evidence for its usage in children is lacking. This chapter will investigate the current level of validity of the notion of treatment-resistant psychosis in children, as well as outline existing guidelines for diagnosis and therapy in this population, with a particular emphasis on the evidence base for clozapine in this cohort.

## 2. The construct of childhood-onset psychosis

DSM-V uses positive symptoms like delusions and hallucinations as well as disorganization in speech and behavior and negative symptoms to diagnose cases of Schizophrenia (4). Hallucinations and delusions are clinically frequently used to diagnose psychosis. But these symptoms are not exclusive to psychosis; they can also be found in trauma, seizure disorders, and other medical illnesses as well as other psychiatric condition of mood disorder and dissociative disorders. A normal child with a busy imagined life can misinterpret thoughts as real events and insist vehemently that a thought or dream occurred (1), which may the criteria for hallucination and delusion, posing diagnostic challenge.

Psychotic symptoms are difficult to distinguish from symptoms associated with other mental illnesses. The table below lists a few considerations to be kept in mind while making a diagnosis of Childhood Psychosis (1, 5, 6).

Language and cognition deficiencies compromise diagnostic precision in younger age groups. The diagnosis is typically valid for older adolescents, a time when first episodes are widespread. Non-specific symptoms such as anxiety, concentration impairments, and irritability may occur before a psychotic episode. This could result in a misdiagnosis.

### 2.1. Schizophrenia prodrome and psychosis-risk syndrome

The emergence of psychotic symptoms in schizophrenia is preceded by a period of mood, perception, cognitive, and social decline that can span from weeks to years. This is especially true for children and adolescents (7). Under the “At-Risk Mental State (ARMS).” Three groups have been identified.

(i) Brief Limited Psychotic Symptoms [BLIPS]- patients experiencing transient psychotic symptoms.
(ii) Attenuated Psychotic Symptoms [APS]- patients with sub-threshold Psychotic symptoms of recent onset.
(iii) Genetic Risk Plus Functional Decline- people who have a first-degree relative (FDR) with schizophrenia and who are now experiencing a deterioration in their functional capacity, or with a diagnosis of schizotypal personality disorder.

Commonly utilized instruments include Comprehensive Assessment of “At- Risk Mental States” (CAARMS) and Structured Interview for Prodromal Symptoms (SIPS).

### 2.2. Prevalence of childhood psychosis as compared to childhood onset mood disorder

Epidemiological evidence implies childhood psychosis is infrequent. Schizophrenia starting in mid-to-late adolescence is 1 percent, whereas Childhood Onset Schizophrenia (COS) is 0.2–0.4 per 10,000 people (8). A landmark study on COS included 1,400 country-wide referrals during a 10-year period (9) where 71 patients met COS criteria at study entry, and 54 children (76%) retained the diagnosis at 10 years. Thus, reflecting that about a quarter of children do not retain the diagnosis after 10 years.

When it comes to affective disorder, 1% of kids and 5% of adolescents suffer from MDD, while 1–2% of people are diagnosed with BPAD. Thus, the prevalence of a pediatric mood disorder is more common.

### 2.3. Do psychotic symptoms imply a diagnosis of schizophrenia?

Childhood psychotic symptoms may be linked to other mental disorders than to schizophrenia (e.g., MDD, BPAD, or dissociative states) (10). A child with a bipolar episode may have hallucinations, delusions, mania, depression, or both. Children with schizophrenia show more formal thought disorder, negative symptoms (e.g., apathy and social withdrawal), and violence (10). Autism spectrum disorders (ASD) can present with oddities in thought like schizophrenia (e.g., the idea that they can talk to their favorite inanimate toys) (11, 12). Misdiagnosis of psychosis can occur with milder forms of autism, like Asperger's syndrome because of unusual perspectives, social difficulties, and concrete brain processes (12) (Table 1). Psychotherapeutic and/or social therapy can relieve psychotic symptoms in children with traumatic hallucinations (usually coupled with nightmares and trance-like states) (11).

**Table 1:** Phenomenology in other childhood diagnosis mimicking psychosis.

**Sl. No**.	**Primary childhood diagnosis**	**Phenomenology that can present mimicking psychosis**
1	Pervasive Developmental Disorder (PDD)	Perseverative thoughts with social skill deficits
2	Attention deficit hyperactive disorder (ADHD)	Behavioral disorganization
3	Obsessive compulsive disorder (OCD)	Severe obsessive thinking
4	Anxiety disorder or childhood PTSD	Severe anticipatory anxiety and traumatic hallucination coupled with nightmares
5	Childhood mood disorder	Inflated self-esteem or irritable mood in association with perceptual abnormalities

#### 2.3.1. Medical conditions presenting with psychosis

There is a wide range of medical diseases associated with psychotic symptoms. Lesions of the central nervous system, whether from disease, trauma, or drug abuse, can trigger psychosis (13). The detailed list of Medical Diagnoses related to Psychotic Episodes is beyond the scope of this chapter. However, before making a diagnosis of primary psychotic disease, it is necessary to rule out secondary causes through a comprehensive medical evaluation.

### 2.4. Phenomenology of childhood psychosis

- Hallucinations and delusions are common in youngsters. Psychotic symptoms aren't a benign occurrence, but they don't always indicate schizophrenia (10).
- Other mental disorders (e.g., depression, anxiety, ADHD, posttraumatic states, and autism spectrum disorders) or medical conditions can cause psychotic symptoms in children and adolescents (10). Prodromal signs often precede psychosis onset.
- For the pharmacological therapy of psychotic symptoms in children, the clinician should choose the proper medication and dose, and then monitor therapeutic results and adverse effects.

### 2.5. Development and cognition associated with psychosis in children

#### 2.5.1. Neurodevelopmental delays

COS children usually exhibit early developmental difficulties. Subtle motor impairments, hypo/hypertonia, sensory processing and integration difficulties, and language delays (14). Pervasive Developmental Disorder (PDD) is indicated by hand flapping, persistent smelling, and touching. Attention difficulties, distractibility, and executive functioning impairment may signal a poor prognosis. Developmental delays and cognitive deficits are seen in schizophrenia spectrum disorders. COS children often have expressive and receptive language impairments that cause cognitive disorder and disorganization (14).

#### 2.5.2. Neurocognition

Literature states children with childhood-onset schizophrenia (COS) have lower IQs than normal children (14). Lower IQ scores possibly reflect a prior neurodevelopmental disorder in such children. Children with schizophrenia often have concentration, memory, and movement problems (14). MRI studies found a link between hippocampal volume and raw information subtest score, possibly reflecting a neuro-biological co-relate for impairment in cognitive function. A decrease in IQ is reported in children with COS during their adolescent period. It is described to be probably due to their inability to master new abilities and is not a regression in pre-existing cognitive reserves. The correlation of schizophrenia with IQ is however highly debated.

### 2.6. Assessment and diagnosis

#### 2.6.1. Screening instruments

Screening instruments can assist discover psychiatric diseases linked with psychotic symptoms before a formal diagnosis is made. Screening instruments in children and adolescents include the Brief Psychiatric Rating Scale for Children (BPRS-C) for psychosis. Children's Depression Inventory (15) for depression and Young Mania Rating Scale (C-YMRS) for mania.

#### 2.6.2. Semi-structured diagnostic interviews

Standardized diagnostic instruments are useful for a number of reasons, including: establishing diagnostic stability; examining symptom dimensions; defining clinical characteristics; and stating predictive validity.

The Schedule for Affective Disorders and Schizophrenia -Present and Lifetime Version (K-SADS-PL) (16), the Diagnostic Interview for Children and Adolescents (DICA) (17), and the Diagnostic Interview Schedule for Children (DISC) (18), are reliable measures for diagnosing MDD, BPAD, COS, and other psychiatric disorders.

#### 2.6.3. Rating scales

Rating scales can help assess psychotic illness symptoms during therapy and over time once a diagnosis has been made. The Child Depression Rating Scale (CDRS) (19) and the Kiddie Version of the Positive and Negative Symptoms of Schizophrenia (K-PANSS) (20) are both effective pediatric COS rating scales. The Clinical Global Impressions-Severity and Improvement (CGI-S and CGI-I) scales, as well as the Clinical Global Assessment of Function (CGAF), assess psychiatric impairment at diagnosis and over time.

### 2.7. Course and prognosis of psychosis in children and adolescents

The symptoms of psychosis in children tend to fluctuate widely over time. However, they might also be a signs of an evolving syndromal psychotic disorder. A birth cohort (10) (*n* = 761) was administered structured diagnostic interviews at ages 11 and 26 to assess symptom prediction. The group was split based on hallucinations or delusions, their severity, and other psychotic symptoms. No child had COS. By the age of 26, those with core symptoms were 16 times more likely to have schizophreniform disorder, and 90% of them had adult occupational and social problems. Even those with mild symptoms were more likely to have adult schizophreniform illness. Forty-two percent of schizophreniform individuals diagnosed at age 26 had weak or significant symptoms at age 11.

An insidious onset pattern is more common in young children before the age of 11 or 12 and has a poorer outcome (5).

Childhood Onset Schizophrenia (COS) is severe, impairs development, and has a poor prognosis. Sixty-seven percent of COS had premorbid social, motor, and language problems, learning impairments, and mood or anxiety disorders (5). Before psychotic episodes, 27% of COS met Autism/ASD criteria. Developmental abnormalities predict prognosis and outcome (5, 21). The consequences of a wrong diagnosis of COS might be severe. Although psychotic symptoms are common (up to 5%) in otherwise healthy youngsters, a diagnosis of Schizophrenia is unlikely. About 30–50% of kids with affective or atypical symptoms are given a COS diagnosis when they shouldn't be, and 90% of kids with a COS diagnosis end up getting another diagnosis (22).

### 2.8. Management of psychosis in children and adolescents

There are three types of childhood psychosis that require different approaches to treatment:

(i) Childhood schizophrenia,
(ii) Depression with psychotic symptoms, and
(iii) Psychosis associated with bipolar affective disorder.

Substance-induced psychosis, medical-related psychosis, and delirium are all treated with antipsychotic medications. In cases where diagnosis is uncertain, like Psychosis NOS, that cannot be properly understood as schizophrenia or a mood illness, it important to wait and watch with close monitoring and offering psychological therapies and social intervention, however in case of severe aggression or agitation neuroleptic and other psychotropic uses should be instituted promptly.

#### 2.8.1. Treatment of childhood onset schizophrenia (COS)

The second -generation antipsychotics are the mainstay of therapy because they cause fewer extrapyramidal symptoms in COS. The Cochrane review of six clinical trials with 256 children and adolescents found limited data to support one antipsychotic medication over another for treating EOS (23). According to the study “Treatment of Early-Onset Schizophrenia Spectrum (TEOSS),” second generation antipsychotics were equally effective in symptom improvement as first generation antipsychotics; clinicians need to be careful about the metabolic side effects of Risperidone and olanzapine in long term follow up (24).

#### 2.8.2. Treatment of psychosis related to pediatric bipolar disorder

Risperidone showed improvement in both psychotic and mood symptoms (25). Use of Olanzapine in children with mania is promising (25). Because atypical antipsychotics have shown good antimanic efficacy in adults, we anticipate similar results for their use in pediatric BPAD.

Divalproex, lithium, and carbamazepine exhibited adequate clinical responses in an open label trial of 42 children and adolescents (aged 8–18 years) with BPAD (25).

#### 2.8.3. Treatment of major depression with psychotic features in childhood

We could not find any open-label study to propose any recommendation for treating psychotic depression in children. A combination of an SSRI like fluoxetine or sertraline in combination with an atypical antipsychotic like olanzapine or risperidone appears to be the rational pharmaceutical approach to treat psychotic depression (26). NICE guidelines recommend intensive psychological therapies along with fluoxetine, augmented with a second-generation antipsychotic medication as the treatment of psychotic depression in children (1, 27).

## 3. Treatment resistant psychosis in children and adolescents

Treatment Resistant Schizophrenia (TRS) is defined as no significant improvement in positive symptoms following treatment with two non-clozapine antipsychotics at adequate dose, duration, and adherence (28).

The landmark study, Treatment of Early Onset Schizophrenia Spectrum Disorders (TEOSS), concluded that more than half of the study participants did not show improvement after 8 weeks of therapy (24).

According to studies on individuals with schizophrenia, response rates to subsequent non-clozapine antipsychotic medication are lower for patients whose disease did not improve with the first antipsychotic. Up to 30% of chronic schizophrenia patients fulfill criteria for TRS, failing to react to 2 non-clozapine antipsychotics (28, 29). Childhood Schizophrenia studies are scarce. Genetic and imaging correlates show childhood-onset schizophrenia has been a continuum with adult-onset schizophrenia, with a persistent, treatment-resistant illness of insidious onset and chronic morbidity (28). Thus, the criteria used for adults with TRS has been extended and used for studies with TRS among children as well.

Contrary to the agreement on primary features of the TRS definition, guidelines differ on definitions of a lack of treatment response and the target clinical outcome measures for treatment response. The concept of pseudo-resistance can complicate determining treatment response (28). Medication non-adherence, pharmacokinetic issues (e.g., drug-drug interactions), and other comorbidities (e.g., substance use disorders, and other medical conditions) might cause pseudo-resistance. This may be more relevant for childhood schizophrenia.

TRS patients who don't respond to clozapine with good compliance or Electro-convulsive Therapy (ECT) are a subset; these are clozapine-resistant and ECT-resistant schizophrenia. No clear criteria exist for treatment resistance psychosis in childhood onset schizophrenia.

## 4. Clozapine in children and adolescents

### 4.1. Clozapine use in treatment-refractory schizophrenia

Very early onset schizophrenia (VEOS) comprises 1% of schizophrenia diagnoses, however they are at significant risk of mortality and disability if treatment is not given efficiently and judiciously. About half of patients with very early-onset schizophrenia (VEOS) do not show adequate clinical response to first line antipsychotics, where clozapine may be useful (23). Despite clozapine's better efficacy for TRS, strangely it has not received not approval from FDA for children and adolescents. Clozapine is the only medicine that helps EOS with treatment resistance, with a better efficacy compared to Olanzapine or Halopreidol (23, 30). Additionally, clozapine has a far reduced risk of extrapyramidal symptoms (EPS) than other antipsychotics. After trying two antipsychotics for 6–8 weeks without improvement, clozapine can be tried. Studies show clozapine is usually utilized after three antipsychotics trial (23). When clozapine is indicated, the hesitancy on the part of psychiatrists for a very rare side effect of bone marrow suppression. there is lack of the lack of substantial evidence-base in children and adolescents regarding the idiosyncratic side effect of agranulocytosis (31).

In past double blind studies, FDA-approved antipsychotic medications such as olanzapine, haloperidol, and quetiapine have been compared with clozapine. Clozapine outperformed quetiapine and risperidone in terms of positive and negative symptom treatment as well as adherence (23). The addition of aripiprazole to clozapine medication may be beneficial in adolescent schizophrenia. Clozapine is recommended for first-episode psychosis (FEP) patients who have not responded well to first-line antipsychotic medications. If patients are not adhering to first-line antipsychotic medications due to side effects or a lack of symptomatic control of psychotic symptoms, initiating Clozapine can be considered (23). Patients with FEP who fail a first-line drug trial are more likely to fail a second drug trial. Given this information and clozapine's demonstrated superiority over other antipsychotics in the treatment of EOS (23), Tungaraza et al. proposed that clozapine be used as a second-line treatment (30, 32). Clozapine significantly reduced hospitalization, suicidality, and substance abuse. Clozapine is usually chosen as the fourth medication, according to studies, and it takes an average of 19.5 months to begin clozapine therapy (30, 32). Early clozapine treatment reduces suicidal behavior and improves cognitive and behavioral outcome.

### 4.2. Review of evidence of clozapine use in children and adolescents

Few randomized controlled trials on clozapine in children exist, but each provides high level of evidence (33–36) (Table 2).

**Table 2:** Review of evidence of clozapine use in childhood psychosis.

**References**	**Drugs**	**Key findings**	**Remarks on side effects**
Kumra et al. (33)	Haloperidol vs. clozapine	Clozapine outperforms haloperidol in terms of both positive and negative symptom control.	Sedation, drooling, and temporary neutropenia were the reported side effects.
Shaw et al. (34)	Olanzapine vs. clozapine	Clozapine showed superior response in mitigating negative symptoms, as measured by SANS score as compared to Olanzapine	Despite reports of increased heartrate and Blood Pressure and enuresis with clozapine, no children were removed from the trial. They found tachycardia in 70% of patients, but no change in blood pressure.
Sporn et al. (35)		NDMC, a clozapine metabolite, was favorably linked with clinical improvement when examined as an NDMC/clozapine ratio	Increased side effects in youngsters than adults, but they could not predict these events.
Kumra et al. (36)	High dose olanzapine vs. clozapine	Among treatment-refractory patients, clozapine had a 66% response rate vs. 33% for olanzapine.	More advantage at a dose of 30 mg/day Olanzapine.
Kumra et al. (36)	Olanzapine to clozapine switchers	70% of olanzapine-refractory individuals reacted to clozapine in an extended study.	Clozapine can cause a range of metabolic adverse effects.

### 4.3. ADR of clozapine in children and adolescents

Literature states the risk of cardiac and metabolic adverse effects in children taking clozapine is higher than that of the other atypical antipsychotics (aripiprazole, olanzapine, quetiapine, and risperidone) (23, 30). Clozapine related Pediatric and adult pharmacokinetics are similar (23). Clozapine causes higher behavioral problems and white blood cell disorders in children. Despite the gap in reported side effects and the tendency for children to develop metabolic syndrome, children are less likely than adults or elderly patients to encounter any side-event with clozapine (24, 29, 30). Clozapine produces less EPS, neuromotor dysfunction, hyperprolactinemia, and liver damage than other antipsychotics (23, 31, 37). Clozapine-induced cardiomyopathy has led to the discontinuation of the drug (23). In adolescent olanzapine and risperidone causes more weight gain than clozapine according to a study of 51 teens (23, 31). Uncertain side effect profile and incidence of metabolic syndrome, sedation, and drooling are expected with clozapine use (23). However, the available literature is limited to a few case series only; and large, controlled studies are suggested to understand population-based side effects of clozapine.

### 4.4. Long term use of clozapine

Few studies are available on long-term clozapine use in children (30). 96.2% of Korean patients had fewer hospital days after a year of clozapine (23, 31). Nearly 70% of 24-year cohort trial patients were compliant after 2 years of maintenance (23, 31). Other trials claim safety up to a year without major side effects.

### 4.5. Recommendations for clozapine use

A recent systematic review (30) and meta-analysis which included four double-blind RCTs, four open label studies, seven observational studies, and three case reports, stated clozapine was well-tolerated and superior in efficacy to other antipsychotics in both short term and long term use, with improvement that sustained during long term follow up. The side-effect profile included sedation, hyper salivation and weight gain commonly and no fatalities reported (30). Severe adverse effects in adolescents are infrequent and outweighed by benefits. Thus, the following recommendations are made:

a. Clozapine should be used for TRS among Children and Adolescents and in some first-line failure patients.
b. Reluctance in clozapine-initiation has been attributed to the risk perceived by clinicians related to use of clozapine, despite evidence that it's safe in children and adolescents. Thus, robust long term follow up studies need to be designed to increase the evidence on safety of use in this cohort.
c. More research is needed on clozapine use in children to develop a consensus or protocol for identifying the target population, minimizing delay in initiation of clozapine and developing a child specific side-effect monitoring checklist.

## 5. Conclusion

Treatment Resistant Psychosis in children and adolescents poses a challenge in diagnosis and management. A comprehensive clinical history helps clarify diagnostic ambiguities. Majority of patients VEOS who do not respond to first-line anti-psychotics are unlikely to benefit from a second non-clozapine antipsychotic. The hazards of delaying therapy when psychotic-symptoms are not immediately managed with a considerable unwanted rise in morbidity and disability has been highlighted often. When other antipsychotics fail, early access to clozapine improves efficacy in risk–benefit analyses. Clozapine is well-tolerated and superior in efficacy to other antipsychotics in both short term and long term use, with improvement that sustained during long term follow up. Long-term clozapine use in young people increases the risk of metabolic side effects include obesity, insulin resistance, diabetes, and dyslipidemia. These side effects damage life quality, although not as much as untreated psychosis. Clozapine long-term use shows promise but needs additional research.

## Author contributions

JP, JR, and UP contributed to the conception and design of the paper. JP, UP, and SC wrote the first draft of the manuscript. All authors wrote different sections of the manuscript, contributed to manuscript revision, read, and approved the submitted version.

## Glossary

ADHD: Attention-Deficit/Hyperactivity disorders
APS: Attenuated Psychotic Symptoms
ARMS: At- Risk Mental State
ASD: Autism spectrum disorders
BLIPS: Brief Limited Psychotic Symptoms
BPAD: Bipolar Affective disorder
BPRS-C: Brief Psychiatric Rating Scale for Children
C-YMRS: Childrens' Young Mania Rating Scale
CAARMS: Comprehensive Assessment of “At-Risk Mental States”
CDRS: Child Depression Rating Scale
CGAF: Clinical Global Assessment of Function
CGI-S and CGI-I: Clinical Global Impressions-Severity and Improvement
COP: childhood-onset psychosis
COS/EOS: Childhood-Onset Schizophrenia/Early Onset Schizophrenia
DICA: Diagnostic Interview for Children and Adolescents
DISC: Diagnostic Interview Schedule for Children
DSM-V: Diagnostic and Statistical Manual-5th Edition
ECT: Electro-convulsive Therapy
EPS: Extra-pyramidal Symptoms
FDR: first-degree relative
FEP: first-episode psychosis
ICD-11: International Classification of Diseases-11th edition
IQ: Intelligence Quotient
K-PANSS: Kiddie Version of the Positive and Negative Symptoms of Schizophrenia
K-SADS-PL: Kiddie's Schedule for Affective Disorders and Schizophrenia—Present and Lifetime Version
MDD: Major Depressive disorder
NICE: National Institute for Health and Care Excellence
OCD: obsessive-compulsive disorder
PDD: pervasive developmental disorder
PNOS: Psychosis Not Otherwise Specified
SIPS: Structured Interview for Prodromal Symptoms
TEOSS: Treatment of Early-Onset Schizophrenia Spectrum
TRS: Treatment Resistant Schizophrenia
VEOS: Very early onset schizophrenia.
